# A scientometric study of Iran and the world countries' contribution to internal medicine (1996-2019)

**DOI:** 10.22088/cjim.13.3.490

**Published:** 2022

**Authors:** Ali Shabestani Monfared, Ali Bijani, Khadijeh Tahmasbi, Mousa Yaminfirooz

**Affiliations:** 1 *Cancer Research Center, Health Research Institute, Babol University of Medical Sciences, Babol Iran *; 2 *Social Determinants of Health Research Center, Health Research Institute, Babol University of Medical Sciences, Babol, Iran*; 3 *College of Management, University of Tehran, Tehran, Iran*

**Keywords:** Internal medicine, Bibliometrics, Journal article, Iran

## Abstract

**Background:**

Considering the importance of internal medicine and the lack of sufficient scientometric data on the research status of the field in Iran, the study aimed at investigating the state of scientific output in the country compared to the countries in the world.

**Methods:**

This applied research used a scientometric approach. The related *MeSH*-driven terms in "internal medicine" were selected as search phrases and searched in the SCImago database. SPSS and Excel software packages were used for statistical analysis. Geographical mapping was done with Google Maps for depicting country geographical distribution.

**Results:**

Out of all 4,972,258 papers published by 230 countries worldwide in the field, about 60% belonged to the USA and Western European countries. These countries were at top of citation and self-citation counts as well as the h-index indicator. The citations per paper indicator were 27.1 and about 25.1% of total citations were self-citations. A significant positive correlation was found between the number of papers, citation count, and self-citation rate, and h-index in the field (p<0.001).

**Conclusion:**

The research status of internal medicine in developed regions and countries was quantitatively and qualitatively better than that of developing countries, including Iran.

The development of scientific and technological fields is one of the main concerns in the world, especially among the developing countries. Scientometrics provides a powerful tool for evaluating scientific development in different fields as well as comparing research output in scientific domains (1). There are some techniques in scientometrics in which one can quantitatively and qualitatively evaluate the scientific output of countries, research institutes and contributing authors. Some main techniques are scientific mapping, citation analysis, word co-occurrence analysis, and co-authorship networks. Some scientometric indicators such as h-index, citescore and impact factor are at work in identifying the developmental process and predicting the research trends in a certain field (2). Nowadays, scientometrics is used in many scientific fields and the findings of scientometric studies help researchers, research institutes, science policy-makers, and scientific journals in identifying their possible gaps and defects in science production (3). By identifying the status of a certain agent active in science production, the core topics and hot subjects are discovered and the placement of the agent is identified in our competitive world. Scientometric studies can show a road map for scientific development. Some scientometric studies have been conducted in the world. In a study on the research trend in internal medicine, it was found that the top producing country was the USA by publishing 438439 (36.99%) papers worldwide. 

Among the Middle-eastern countries, Turkey and Iran were top-producing countries that contributed to publishing 56% of all papers published in the region (4). Other scientometric studies showed that the USA and European countries are pioneered in other scientific fields (5-11). Sometimes, countries from other regions, such as India (6), Turkey (8), China (9) and Australia (10) have been among the highly-active countries throughout the world. It is argued that high-income countries are at the top of scientific production in the world as they spend more on international research and achieve more economic productivity as a result of their research development (7, 11). Research conducted in the Middle East showed that countries such as Iran, Israel, Saudi Arabia and Turkey have more contributions to scientific development (12-14).

As the main discipline in the medical field, internal medicine deals with the prevention, diagnosis and treatment of internal diseases (15). Internal medicine professionals are active in clinical education and research as they have an extended role in the prevention and treatment of a variety of diseases. 

As Iranian researchers in many scientific fields tend to develop the science landscape in the world, especially in recent years, their mere contribution in individual disciplines has not been deeply investigated from a scientometric perspective. Some medical fields, including among others, internal medicine are of these fields. Considering this gap as well as the importance of internal medicine and the lack of sufficient scientometric data on the placement of the field in Iran, the study aimed at investigating the state of scientific output in the country compared to the countries in the world as well as mapping its scientific networks. 

## Methods

This applied research used a scientometric approach. The related *MeSH*-selected terms in "internal medicine" were selected as search phrases. It was notable that internal medicine was selected as the preferred main entry in *MeSH* with its 10 main headings as follows: 

Internal Medicine [H02.403.429] 

- Cardiology [H02.403.429.163] 

- Cardiac Electrophysiology [H02.403.429.163.300]

- Endocrinology [H02.403.429.323]
- Gastroenterology [H02.403.429.405]
- Hematology [H02.403.429.445] 

- Transfusion Medicine [H02.403.429.445.500]

- Infectious Disease Medicine [H02.403.429.480]
- Medical Oncology [H02.403.429.515] 

- Psycho-Oncology [H02.403.429.515.250]
- Radiation Oncology [H02.403.429.515.500]
- Surgical Oncology [H02.403.429.515.750]

- Nephrology [H02.403.429.580]
- Pulmonary Medicine [H02.403.429.675]
- Rheumatology [H02.403.429.730]
- Sleep Medicine Specialty [H02.403.429.865]

These topics were all searched for retrieving the related data. Using the SCImago database, all related items were retrieved from it based on the "subject categories section" in the Country Ranking box and saved in Excel. As the subheading "sleep medicine specialty" did not appear in SCImago, the topic was excluded. For avoiding possible overlap, general internal medicine was not considered as a separate category. 

All retrieved data were checked based on a researcher-made checklist. All papers published during 1996-2019 in the field were considered as the study populations. SPSS and Excel software packages were used for statistical analysis. Geographical mapping was done with Google Maps for depicting country geographical distribution.

## Results

**Figure 1:**
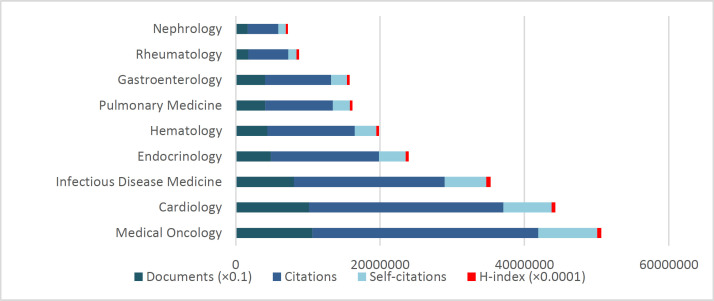
Some comparative scientometric indicators of world's countries in publishing on the subfields of internal medicine

Table 1 and figure 1 show the publication indicators related to all 230 countries publishing in 9 studied subfields of internal medicine. These papers amounted to 4,972,258 with oncology subfield at the top (N=1061373) and nephrology at the bottom (N=168456). The mean rate of citations per paper indicator (CPP) was 27.1, with rheumatology in the highest rank (31.7) and gastroenterology in the lowest rank (22.6). 

The oncology subfield had the first rank in receiving citations (N=31306796), followed by cardiology (N=26937440) and infectious disease medicine (N= 20874363) subfields. Considering self-citation rates, the highest and lowest rates belonged to internal medicine (27.6%) and rheumatology (20.2%), respectively. In total, 25.3% of citations were self-citations. 

Considering h-indices, the first to third ranks belonged to infectious disease medicine (59.1), medical oncology (55.4), and cardiology (52.2), respectively. 

**Table 1:** Some scientometric indicators of world's countries in publishing on the subfields of internal medicine

Subfield	Documents	Citations	Citations per Paper	Self-citations **(%)**	H index** (Mean)**
**Medical Oncology**	1061373	31306796	29.5	26.1	55.4
**Cardiology**	1015297	26937440	26.5	24.8	52.2
**Infectious Disease Medicine**	808275	20874363	25.8	27.6	59.1
**Endocrinology**	488300	14973421	3.7	24.7	43.3
**Hematology**	440244	12108966	27.5	24.5	38.9
**Pulmonary Medicine**	410061	9359045	22.8	25.4	35.2
**Gastroenterology**	405316	9179325	22.6	23.7	38.6
**Rheumatology**	174936	5548008	31.7	20.2	36.4
**Nephrology**	168456	4212395	25.0	24.8	3.4
**Total**	4972258	134499759	27.1	25.3	43.3

**Figure 2:**
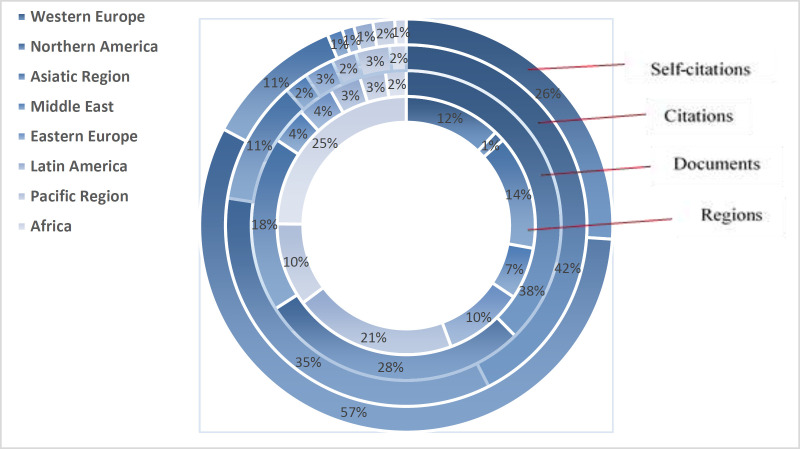
Contribution of different regions in producing scientific output of internal medicine based on document number, citation count and self-citation rate

**Figure 3:**
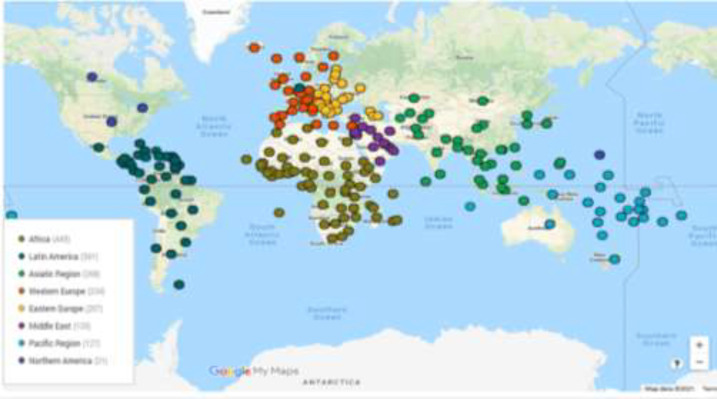
Distribution of world's regions/countries based on publishing scientific papers on internal medicine

**Figure 4:**
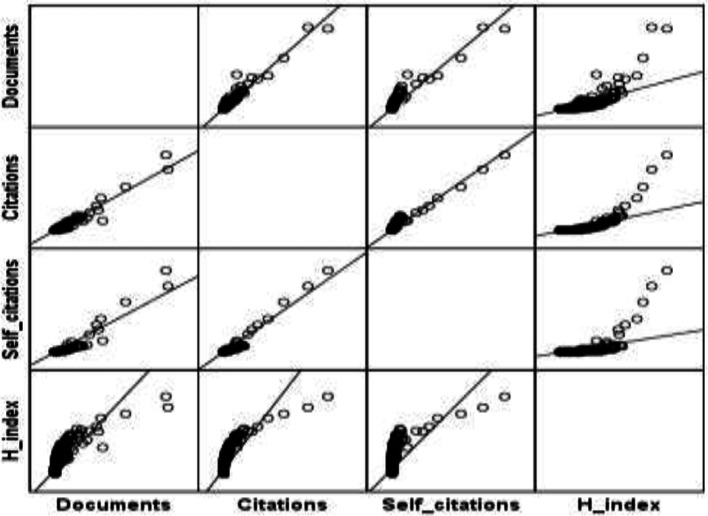
Correlation matrix between the number of papers, citation counts, self-citation counts and h-index rates in some scientometric indicators of internal medicine

Figure 2 depicts the amounts of contributions made by the world's regions in internal medicine. As can be seen, Western Europe ranked first with publishing 38% of total papers published by 28 Western European countries. The second and third ranks belonged to the Northern American countries (with 28% in total) and Asian countries (with 12% in total), respectively. Other regions contributed to 16% of total published papers in the field. Table 2 shows some main scientometric indicators of the top 30 countries in publishing papers on internal medicine. The top three countries in publishing papers on internal medicine were the USA (with 25% of published papers), Japan (with 6.6% of published papers) and the UK (with 6.5% of all published works), respectively. The top three highly influential countries calculated by their received citation counts were the USA, the UK and Germany. However, the top three high ranked countries in their CPPs were Finland (40.5), Belgium (37.6) and Sweden (37.1), respectively. In self-citation, the USA (with 44.8%), China (42.4%) and Iran (35.6%) were the three top-ranked countries, respectively. The 25^th^ rank in publishing on internal medicine belonged to Iran as can be seen in table 3. With publishing 32,495 papers on the field, Iran ranked third in the Middle East, after Turkey (12^th^) and Israel (24^th^). The country was 3^rd^ ranked in self-citation worldwide and first-ranked in the Middle East. For studying the possible relationship between the number of papers, citation counts, self-citation counts and h-index rates, Pearson's correlational test was conducted in the internal medicine field. As table 3 shows, there is a significant positive relationship among the studied variables (p<.01). However, the relationship between self-citation counts and h-index rates was at a moderate level. The highest correlated variables were the number of papers and received citation counts (r=.973). The highest correlation was seen in the cardiology subfield in this regard (r=.991). Figure 4 depicts the correlation matrix. Figure 3 mapped the geographical distribution of world's regions/countries based on publishing scientific papers on internal medicine. 

**Table 2:** Some main scientometric indicators of top countries in publishing papers on internal medicine

**Rank**	**Country**	**Region**	**Documents**	**Citations**	**Citations per Paper**	**Self-citations(%)**
1	United States	Northern America	1241238	41184078	33.2	44.8
2	Japan	Asiatic Region	327709	6213638	19.0	24.5
3	United Kingdom	Western Europe	324613	10697373	33.0	18.9
4	Germany	Western Europe	287546	8144690	28.3	19.5
5	China	Asiatic Region	275070	3183074	11.6	42.4
6	Italy	Western Europe	263489	6895930	26.2	19.3
7	France	Western Europe	234742	6525575	27.8	15.8
8	Canada	Northern America	170118	5868360	34.5	14.1
9	Spain	Western Europe	144221	3421399	23.7	16.0
10	Netherlands	Western Europe	141628	5174257	36.5	13.8
11	Australia	Pacific Region	116691	3603530	30.9	14.8
12	Turkey	Middle East	85656	924280	10.8	17.0
13	India	Asiatic Region	84327	994367	11.8	25.6
14	South Korea	Asiatic Region	83802	1450491	17.3	16.0
15	Brazil	Latin America	80228	1335800	16.7	24.9
16	Switzerland	Western Europe	79548	2834170	35.6	9.7
17	Sweden	Western Europe	77397	2872981	37.1	11.8
18	Belgium	Western Europe	66284	2493395	37.6	9.6
19	Poland	Eastern Europe	60242	925805	15.4	13.2
20	Denmark	Western Europe	52116	1911659	36.7	11.8
21	Greece	Western Europe	49154	1153644	23.5	10.4
22	Taiwan	Asiatic Region	45088	940605	20.9	15.7
23	Austria	Western Europe	43891	1392331	31.7	8.8
24	Israel	Middle East	37820	1158624	30.6	8.2
25	Iran	Middle East	32495	299995	9.2	35.6
26	Finland	Western Europe	29219	1183119	40.5	9.6
27	Czech Republic	Eastern Europe	29041	519620	17.9	10.5
28	Norway	Western Europe	28868	1056155	36.6	9.3
29	Russian Federation	Eastern Europe	28176	318179	11.3	11.1
30	Mexico	Latin America	22212	380191	17.1	13.1

**(** https://www.google.com/maps/d/u/0/edit?mid=1rKj7ZlzwQBBL-hSE9tB_x68dNe9rXfPH&ll=2.45746631940213%2C0&z=2 **)**

**Table 3:** Pearson's correlation coefficients for testing relationship between the number of papers, citation counts, self-citation counts and h-index rates in internal medicine

Subfields	Documents	Documents	Documents	Citations	Citations	Self-citations
	Citations	Self-citations	H-index	Self-citations	H-index	H-index
**Cardiology**	0.991	0.951	0.752	0.965	0.738	0.563
**Gastroenterology**	0.956	0.913	0.766	0.961	0.769	0.593
**Hematology**	0.990	0.927	0.805	0.949	0.778	0.587
** Endocrinology**	0.987	0.936	0.810	0.964	0.769	0.608
**Infectious Disease Medicine**	0.986	0.941	0.796	0.969	0.752	0.609
**Nephrology**	0.985	0.93	0.812	0.968	0.76	0.602
**Medical Oncology**	0.961	0.924	0.767	0.969	0.75	0.595
**Pulmonary Medicine**	0.983	0.942	0.766	0.963	0.753	0.581
**Rheumatology**	0.982	0.92	0.836	0.942	0.824	0.636
**Total**	0.973	0.929	0.736	0.965	0.712	0.551

Correlation is significant at the.01 level (2-tailed).

## Discussion

This study included the scientific output made by 230 countries on internal medicine produced during 1996-2018 and indexed in the SCImago database. We found that the majority of papers (58%) considered topics on oncology, cardiology, and infectious diseases. Various causes can be considered in this regard. One is the prevalence of some diseases in the world, resulting in producing more papers on some topics related to these diseases. Some studies introduced cancers (16) and cardiovascular diseases (17) as the main causes of mortality. It can be said that there is a direct relationship between the prevalence of a certain disease worldwide and research on it and consequent more scientific production about the disease (18). Considering the geographical distribution of producing regions on internal medicine, it was found that the USA and Western Europe contributed to about 60% of total output. Western Europe was the top producing region in rheumatology (19). However, Northern America was more productive than Western Europe in traumatic brain injury rehabilitation (3), cardiovascular and thoracic research (20) and pneumonia research (21). In addition, the USA was in the top of producing countries in internal medicine with contributing to 25% of papers, followed by Japan as an Asian country. In other medical fields, the USA was at top of producing countries (16-21). Some Asian countries, including Japan, China, India, South Korea and Taiwan had better performance in internal medicine compared to some Middle Eastern and European countries. Such a finding was obtained in homeopathy (6), rheumatology (19) and pneumonia (21). Among the Middle Eastern countries, Turkey, Israel and Iran ranked first to third respectively. These countries were top in endocrinology research (12). In general, internal medicine (13), Turkey and Iran ranked first and third, respectively. Iran ranked second in thyroids research (14). In spite of their world rankings, these countries have better rankings in the region. 

The mean rate of CPP in internal medicine was 27.1%. of them, 25.1% were self-citations. Among the countries, the USA, the UK and Germany were top highly-cited and Iran was less-cited countries. In calculating the ratio of citations to papers, the top countries were Finland, Belgium and Sweden and Iran ranked the last. It can be concluded that high scientific productivity cannot be the sign of high scientific influence as seen by (19). In the case of Iran, some studies found that the country has low rates in citations per paper (12, 13) and it can be a sign of its rather low-quality papers in some fields, such as internal medicine. Self-citation with its various motivations –such as promotion, more citability and grants- is manifested together with citation (22, 23). If used too much, self-citation becomes problematic. Self-citation rate more than 20% of total received citation is conceived as illegible scientific action (24). We found that the mean rate of self-citations in all subfields of internal medicine was higher than the expected rate, especially among top producing countries as well as Iran. Despite its low rank in receiving citations, Iran ranked third in the self-citation indicator after the USA and China, with 35.36% in self-citation. Similar findings can be seen in other studies in which these three countries were top in self-citation rates (25, 26). Some reasons direct citation patterns, including orientation toward authors of the same country as can be seen in cases of the USA and the UK (27). Geographical placement, cultural communication, language similarity and so on affect citation patterns (28). However, one possible reason for top countries' self-citation is their collaboration and common research projects in intra-region levels (25). 

The results showed that there is a significantly positive correlation between the number of papers, citation counts and self-citation rate. As noted in other studies (25, 29), these measures are at work in evaluating the scientific performance of countries, research institutes, journals and authors. However, these factors encounter some challenges in quantifying and qualifying the scientific state. A comprehensive indicator is needed to be developed for quantification and qualification of science, as highlighted in the h-index (30). We found that there is a positive relationship between the number of papers and the h-index as well as citation counts and the h-index. As a result, a change in these variables affects h-index rates. In addition, the finding is reasonable as h-index is measured by including the citation count and paper number. Some studies confirmed such a relationship (31, 32). 

We found a moderated relationship between self-citation count and h-index. However, some argued that h-index is hardly affected by the self-citation rate (33) and others concluded that removing self-citation counts has low effect (<1) on h-index rate (34). In addition, a study found that self-citation count had no effect on country rankings of 58% of countries, positive effect on the country ranking of 26% of countries and negative effect on that of 16% of countries (25). In another study, it was found that if excluded from total citations, self-citation results are in a considerable decrease in h-index (35). Nowadays, despite the ever-increasing development of information production and development, a complex and confusing scientific sphere has been dominated. Scientometric studies in different fields help researchers in finding their ways for better contribution and collaboration in research and consequently, producing high-quality scientific output. Like any other quantitative/ dcientometric studies, the study has some limitations. One main is that indexing/abstracting databases, including SCImago database, have their own limitation as to their inclusion and scope (36). However, they can logically depict the scientific output from bibliometric aspects. This article is the first to study Iranian researchers' activity in contribution to internal medicine region-wide and worldwide. 
